# Study of the Hypoglycemic Activity of Derivatives of Isoflavones from* Cicer arietinum* L.

**DOI:** 10.1155/2017/8746823

**Published:** 2017-03-21

**Authors:** Ying Wei, Pengshou Li, Bo Li, Jiaqi Gao, Dongchao Wang, Lingling Qin, Wen Sun, Yunling Xu, Haoxia Shi, Tunhai Xu, Tonghua Liu

**Affiliations:** ^1^School of Chinese Pharmacy, Beijing University of Chinese Medicine, Beijing, China; ^2^Health Cultivation Key Laboratory of the Ministry of Education, Beijing University of Chinese Medicine, Beijing, China; ^3^Health Cultivation Key Laboratory of Beijing, Beijing University of Chinese Medicine, Beijing, China

## Abstract

The chickpea, a food and medicine used by the people of Xinjiang, has a beneficial hypoglycemic effect. To better utilize this national resource and develop hypoglycemic agents from components of the chickpea, a series of new derivatives of isoflavone compounds from the chickpea were synthesized. An insulin-resistant (IR) HepG2 cell model was used to screen the hypoglycemic activities of these compounds. And the structure-activity relationships of these compounds were explored. Additionally, several combinations of these compound displayed higher hypoglycemic activity than any single compound, and they had similar hypoglycemic activity to that of the positive control group (*p* > 0.05). In addition, combination 3 and combination 6 exerted different effects on the insulin sensitivity of H4IIE cells stimulated with resistin. And the results indicated that combination 3 would have higher hypoglycemic activity. These findings demonstrate the characteristics of multiple components and targets of Chinese herbal medicine. This evidence may provide new ideas for the development of hypoglycemic drugs.

## 1. Introduction

The chickpea (Cicer arietinum L.) is a leguminous herb originating from western Asia and the Near East that has been widely introduced throughout the world. The chickpea is an important vegetable in India and Pakistan and is frequently eaten in Europe as well. It is also commonly used in traditional Uighur medicine. The chickpea is rich in many vitamins and minerals. The chickpea has obvious effects such as blood enrichment and calcium supplementation, and it is the best food for patients with diabetes [1–4]. The chickpea is primarily distributed in Xinjiang, Qinghai, Gansu, and other provinces in China. Mulei County in Xinjiang is the area with the greatest chickpea production. There is a 2000-year history of the use of the chickpea as a drug in the Xinjiang area, and the chickpea has been used for the treatment of diabetes in traditional Uygur medicine and traditional Chinese medicine.

In the daily diet of the Uygur people in Xinjiang, beef and mutton, milk, and fruits with high sugar contents are the staple foods. However, few people suffer from diabetes and cardiovascular diseases despite their intake of foods high in fat, calories, and protein. The World Health Organization found that the Uygur people's staple food of hand pilaf mixed with chickpea is the key to a balanced meal and prevents the development of diseases associated with conditions such as high blood pressure, high blood sugar, high blood lipids, and diabetes. Modern research has found that the isoflavone fraction from chickpeas has hypoglycemic activity [5–8], demonstrating that the chickpea is a natural product with hypoglycemic activity. Further research has shown that although these components have hypoglycemic activity, their activity is not very high. Therefore, to produce compounds with higher hypoglycemic activity than these chickpea components, the structures of monomeric chickpea compounds must be modified to optimize the physical and chemical properties of the compounds.

In this study, genistein, biochanin A and fermononetin were subjected to structural modification. They are the main isoflavones in chickpeas. Genistein has clear hypoglycemic activity, but its application is limited [9, 10]. This may be due to the chemical structure of this isoflavone and phenolic hydroxyl moiety, which is responsible for its low bioavailability. The study of formononetin has been focused on its role in auxiliary hypoglycemic drugs, and it does not appear as the main active ingredients in any hypoglycemic drug [11]. There were a few reports of the hypoglycemic activity of biochanin A, and its hypoglycemic activity was not strong [12]. These findings hindered the development and utilization of isoflavones from chickpeas. Structurally modifying these compounds could optimize their properties, thus increasing their hypoglycemic activity, and could promote the development and utilization of isoflavones produced by chickpeas.

In recent decades, drug discovery has focused on identifying and designing highly selective ligands for individual targets. However, it is difficult for a drug to achieve its desired effect or to exert toxicity on a single target in the treatment of a disease. It is also difficult to cure many diseases, such as cancer or diseases affecting multiple tissues or cells, including diabetes [13]. Moreover, single-target drugs not only rarely exert the desired treatment effect but also even provoke adverse reactions. The combination of several different single-target drugs and the use of multiple-target drugs would produce enhanced therapeutic effects for the treatment of complex diseases [14, 15]. A multipronged treatment strategy could overcome the limitations of many single-target drugs. Multitarget therapeutics have been used in the treatment of major diseases [16]. Multicomponent drugs are becoming increasingly used to treat type 2 diabetes, such as Glucovance and Avandamet [17]. These multicomponent drugs can stabilize blood glucose levels by adjusting the target in multiple tissues.

**Figure 1:**
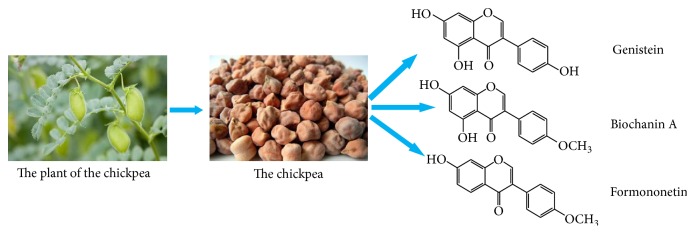
The chickpea and its three isoflavones.

In addition, taking into account the characteristics of many effective ingredients and multiple targets of Chinese herbal medicine, combining multiple compounds could result in enhanced hypoglycemic activity. Therefore, the above three isoflavones produced by chickpeas were subjected to many structural modifications, and the hypoglycemic activity of these derivatives was screened. Then, the compounds with good hypoglycemic activity were selected for mixture into combinations, and the hypoglycemic activity and other aspects of these combinations in diabetes were studied. These studies would be beneficial to the development and utilization of isoflavone from chickpea. It also provides some references for the research and development of hypoglycemic drugs. The chickpea and its three isoflavones are shown in Figure 1.

## 2. Materials and Methods

### 2.1. Materials and Instruments

Nuclear magnetic resonance (NMR) detection: Bruker AV500 MHz spectrometer (Germany); high-resolution mass spectrometric (HR-MS) detection: Thermo Scientific LTQ Orbitrap XL mass spectrometer (New York, NY, USA); thin-layer chromatography (TLC) detection: silica gel GF-254 plates (Qingdao Haiyang Corporation, China); silica gel: 300–400 mesh (Qingdao Haiyang Corporation, China); elemental analysis: Vario EL element analyzer; ultraviolet detection: UV-2450 ultraviolet visible spectrophotometer; melting point detection: X-5 micro melting point apparatus (Beijing, China); infrared detection: Thermo Nicolet Nexus 670 spectrometer (New York, NY, USA). Yields were calculated based on the last step of the reaction. All of the chemicals and solvents that were used were of analytical or high-performance liquid chromatography grade.

### 2.2. Experimental Methods

#### 2.2.1. Chemistry

**Figure 2:**
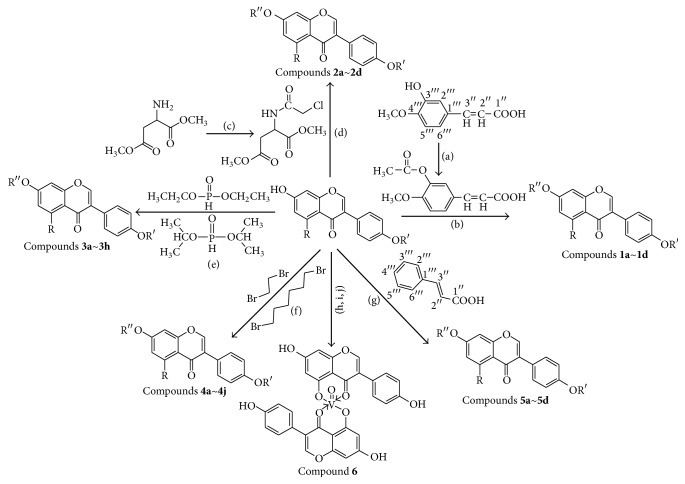
Synthetic routes.* Reagents and conditions*: (a) (CH_3_CO)_2_O, Py, reflux 4 h; (b) DCC, Py, reflux, 80°C, 4 h. (c) ClCH_2_COCl, NaHCO_3_, EA, r.t., 0.5 h; (d) K_2_CO_3_, KI, DMF, 24 h. (e) CCl_4_/Et_3_N, C_2_H_5_OH, 24 h. (f) K_2_CO_3_, CH_3_CH_2_OH, reflux 24 h. (g) DCC, Py, reflux, 80°C, 4 h. (h) CH_3_OH, 60°C, 1 h; (i) VO(acac)_2_, r.t., 0.5 h; (j) stand in air for a few days for crystallization. Parent compounds: R = OH, H; R′ = H, OCH_3_.

The synthetic routes are shown in Figure 2.

Rationale for structural modifications is as follows:

1. Based on the principles of drug design, flatten principle is an important way of structure modification. Considering that isoferulic acid has a strong hypoglycemic activity [18–21], the isoflavones were esterified with acetyl isoferulic acid. This structural modification was expected to improve their efficacy through possible synergy.
2. Evidence shows that amino acids or short peptides split with biologically active molecules could change the nature of the active molecules and enhance their effects and selectivity for target cells. In addition, through molecular condensation, the transmission of the drug across the cell membrane was also enhanced, and its bioavailability was improved [22, 23].
3. Related studies have found that most western medicines contain not only C, H, and O but also other elements, such as N, P, and S, which can increase the affinity for the target point. Therefore, to enrich the types of elements found in compounds from traditional Chinese medicine, the P element was introduced via the structural modification of phosphorylation.
4. In the process of the structural modification of isoflavone compounds from chickpea, most western medicines were found to contain not only C, H, and O but also other elements including N, P, and S. In addition, we performed structural modifications by adding N, P, S and other elements. Bromine has also been applied in many western medicines. However, there is no bromine component in various hypoglycemic drugs. Thus, it was desirable to introduce bromine element into the compounds and study its effect on the hypoglycemic activity of the compounds.
5. A condensation reaction with cinnamic acid was designed because cinnamaldehyde has a strong hypoglycemic effect [24–27]. Thus, the potential synergistic activity of this product may result in enhanced selectivity and hypoglycemic effects.
6. Vanadium is an essential trace element in the human body [28]. Much evidence has shown that diabetic patients have vanadium deficiencies. Supplementation with vanadium compounds can reduce blood sugar. Further study of the mechanism underlying this effect showed that vanadium acts on the insulin receptor directly and increases its insulin sensitivity. The mechanism underlying the hypoglycemic activity of vanadium is unique, and the vast majority of current hypoglycemic drugs do not contain vanadium. This mechanism opens up new possibilities for the research and development of antidiabetic drugs. In recent years, the vast majority of vanadium compounds tested in biological studies of antidiabetic drugs in China and abroad have been simple salts, such as NaVO_3_ and VOSO_4_. These compounds have poor lipid solubility and are difficult to absorb. In addition, these compounds have the shortcoming of gastrointestinal tract irritation. These conditions limit their further application and development [29]. In this study, a new type of organic vanadium complex was synthesized to increase the lipid solubility of vanadium compounds and reduce their toxicity to the gastrointestinal tract.

*(1) General Method for the Synthesis of Compounds* ***1a–1d*** *(Describing One of the Parental Isoflavone Compounds as an Example)*

*Treatment of Acetic Anhydride*. P_2_O_5_ was added to acetic anhydride until saturation of the P_2_O_5_. The mixture was filtered to remove the P_2_O_5_ powder. The filtrate was distilled at 90°C and 0.096 MPa; fractions were collected at a constant temperature and stored in a dryer [30, 31].

*Treatment of Pyridine*. KOH was added to pyridine and refluxed for 5 h until the KOH became a paste. Then, the reflux was stopped to distill the solution at a lower pressure. Fractions were collected at 68°C until the pressure reached 0.085 MPa, and the pyridine was finally sealed with wax [31].

*Synthesis of Acetyl Isoferulic Acid*. A total of 1 g (5.15 mmol) isoferulic acid and 20 mL acetyl chloride was placed in a 100-mL round-bottom flask, and the flask was placed in an ice bath to reduce the temperature of the mixture to approximately 0°C. Then, 5 drops of anhydrous pyridine were added at 0°C. The temperature of this mixture was increased to 120°C under stirring, and the reaction was conducted under reflux for 4h. After the reaction, the mixture was cooled at room temperature, and a large number of solid formed. The solid was filtered and washed with water and anhydrous ethanol. The precipitate was dried to obtain the crude product of acetyl isoferulic acid. The crude material was then purified in a 300–400 mesh silica column (petroleum ether : ethyl acetate : methanol: acetic acid, 30 : 10 : 5 : 1).

The weights of genistein (0.30 g, 1.110 mmol), acetyl isoferulic acid (0.10 g, 0.423 mmol), and DCC (0.4 g, 1.940 mmol) were measured. Then, 20 mL of tetrahydrofuran (THF) and 1.5 mL of anhydrous pyridine were added to this mixture under stirring. The mixture was then stirred at 80°C for 4 h. After the reaction was completed, the resulting mixture was filtered to remove the DCU. THF was vaporized, and ethyl acetate was added to this mixture. 1 mol/L HCl was prepared to remove pyridine, and the ethyl acetate layer was collected to obtain crude material, which was then purified in a 300–400 mesh silica column (petroleum ether : ethyl acetate : methanol : acetic acid, 30 : 10 : 5 : 1). The crude product of biochanin A and formononetin were purified in 300–400 mesh silica columns (petroleum ether: ethyl acetate, 6:1). 

*7-Acetyl Isoferulic Acid-O-genistein (* ***1a***). The product was obtained as a white solid (22.5%); m.p. 253.1~253.8°C. ^1^H-NMR (DMSO-*d*_6_) *δ* (ppm): 12.91 (s, 1H, 5-OH), 9.63 (s, 1H, 4′-OH), 8.52 (s, 1H, H-2), 8.02 (d, 1H,* J* = 15.2 Hz, H-3′′), 7.68 (s, 1H, H-2′′′), 7.49 (d, 2H,* J* = 8.1 Hz, H-2′,6′), 7.15–7.36 (m, 2H, H-5′′′, H-6′′′), 6.93 (d, 1H,* J* = 15.3 Hz, H-2′′), 6.85 (d, 2H,* J* = 8.4 Hz, H-3′,5′), 6.52 (s, 1H, H-6), 6.32 (s, 1H, H-8), 3.89 (s, 3H, -OCH_3_), 2.36 (s, 3H, -COCH_3_); ^13^C NMR (DMSO-*d*_6_) *δ* (ppm): 181.34 (C-4), 167.52 (C-1′′), 165.30 (-COO-), 161.82 (C-5), 158.22 (C-8a), 156.19 (C-7), 155.36 (C-4′), 155.09 (C-2), 152.63 (C-3′′′), 145.27 (C-3′′), 140.61 (C-4′′′), 133.52 (C-1′′′), 131.14 (C-2′,6′), 123.87 (C-1′), 123.62 (C-5′′′), 122.13 (C-3), 120.32 (C-6′′′), 117.50 (C-2′′′), 115.89 (C-3′,5′), 112.40 (C-2′′′), 109.89 (C-4a), 105.33 (C-6), 101.89 (C-8), 56.76 (-OCH_3_), 20.81 (-CH_3_). HRMS (ESI)* m*/*z*: 489.12178 [M + H]^+^, calcd. for C_27_H_21_O_9_ 489.11856. 

*4*′*,7-Diacetyl Isoferulic Acid-O-genistein ( ****1b***). The product was obtained as a white solid (15.2%); m.p. 243.2~243.9°C.^1^H NMR (DMSO-*d*_6_) *δ* (ppm): 12.99 (s, 1H, 5-OH), 8.65 (s, 1H, H-2), 7.84 (d, 2H,* J* = 15.64 Hz, H-3′′), 7.71 (s, 2H, H-2′′′), 7.65 (d, 2H,* J* = 8.6 Hz, H-2′,6′), 7.49 (d, 2H,* J* = 8.5 Hz, H-3′,5′), 7.43 (d, 2H,* J* = 8.7 Hz, H-6′′′), 7.28 (d, 2H,* J* = 8.4 Hz, H-5′′′), 7.13 (d, 2H,* J* = 15.8 Hz, H-2′′), 6.89 (s, 1H, H-6), 6.75 (s, 1H, H-8), 3.89 (s, 6H, -OCH_3_), 2.38 (s, 6H, -CH_3_); ^13^C-NMR (DMSO-*d*_6_) *δ* (ppm): 180.79 (C-4), 167.12, 166.41 (s, C-1′′), 164.82, 164.49 (-COO-), 157.12 (s, C-5), 155.79 (C-4′), 153.11 (C-7), 151.93 (C-8a), 151.29, 150.22 (C-3′′′), 149.30 (C-2), 145.60, 145.10 (C-3′′), 142.12, 141.81 (C-4′′′), 132.11, 131.71 (C-1′′′), 129.89 (C-2′,6′), 122.82 (C-1′), 121.61, 121.21 (C-5′′′), 120.51 (C-3), 120.21, 119.32 (C-6′′′), 116.72, 116.09 (C-2′′), 112.80 (C-3′,5′), 110.23, 110.10 (C-2′′′), 104.51 (C-4a), 100.53 (C-6), 95.80 (C-8), 55.31 (two -OCH_3_), 19.81 (-CH_3_), 19.09 (-CH_3_). HRMS (ESI)* m*/*z*: 707.17632 [M + H]^+^, calcd. for C_39_H_31_O_13_ 707.17647. 

*7-Acetyl Isoferulic Acid-O-biochanin A (* ***1c***). The product was obtained as a buff solid (32.1%); m.p. 197.4~198.2°C. ^1^H-NMR (DMSO-*d*_6_) *δ* (ppm): *δ* 13.16 (s, 1H, 5-OH), 8.67 (s, 1H, H-2), 8.22 (d, 1H,* J* = 15.3 Hz, H-3′′), 7.91 (s, 1H, H-2′′′), 7.62 (d, 2H,* J* = 8.3 Hz, H-2′,6′), 7.41–7.56 (m, 2H, H-5′′′, H-6′′′), 7.17 (d, 1H,* J* = 15.1 Hz, H-2′′), 7.08 (d, 2H,* J* = 8.2 Hz, H-3′,5′), 6.61 (s, 1H, H-6), 6.41 (s, 1H, H-8), 3.95 (s, 3H, -OCH_3_), 3.70 (s, 3H, -OCH_3_), 2.40 (s, 3H, -COCH_3_). ^13^C-NMR (DMSO-*d*_6_) *δ* (ppm): 181.33 (C-4), 168.86 (C-1′′), 166.46 (-COO-), 161.77 (C-5), 159.80 (C-8a), 156.88 (C-7), 156.32 (C-4′), 155.95 (C-2), 151.36 (C-3′′′), 150.75 (C-3′′), 139.66 (C-4′′′), 133.36 (C-1′′′), 130.68 (C-2′,6′), 128.60 (C-1′), 125.70 (C-5′′′), 123.10 (C-3), 122.96 (C-6′′′), 116.64 (C-2′′), 114.25 (C-3′,5′), 111.36 (C-2′′′), 109.24 (C-4a), 105.82 (C-6), 101.85 (C-8), 55.67 (-OCH_3_), 55.03 (-OCH_3_), 21.40 (-CH_3_). HRMS (ESI)* m*/*z*: 503.14521 [M + H]^+^, calcd. for C_28_H_23_O_9_ 503.13421.

*7-Acetyl Isoferulic Acid-O-formononetin (* ***1d***). The product was obtained as a white solid (35.1%); m.p. 241.1~241.9°C. ^1^H-NMR (DMSO-*d*_6_) *δ* (ppm): 8.42 (s, 1H, H-2), 8.03 (d,* J* = 8.6 Hz, 1H, H-5), 7.65 (d,* J* = 16.1 Hz, 1H, H-3′′), 7.61 (s, 1H, H-2′′′), 7.48 (d,* J* = 6.9 Hz, 2H, H-2′,6′), 7.35 (d,* J* = 8.2 Hz, 1H, H-6′′′), 7.24 (d,* J* = 8.3 Hz, 1H, H-5′′′), 7.05 (d,* J* = 8.1 Hz, 1H, H-6), 6.91 (d,* J* = 7.4 Hz, 2H, H-3′,5′), 6.85 (s, 1H, H-8), 6.72 (d,* J* = 16.1 Hz, 1H, H-2′′), 3.85 (s, 3H, -OCH_3_), 3.76 (s, 3H, -OCH_3_), 2.35 (s, 3H, CH_3_COO-).^13^C-NMR (DMSO-*d*_6_) *δ* (ppm): 180.89 (C-4), 168.57 (C-1′′), 164.38 (-COO-), 162.52 (C-7), 156.92 (C-4′), 156.47 (C-8a), 153.48 (C-2), 150.21 (C-3′′′), 146.50 (C-1′′), 141.23 (C-4′′′), 131.99 (C-1′′′), 130.78 (C-2′,6′), 128.51 (C-5), 126.44 (C-1′), 124.56 (C-3), 123.41 (C-5′′′), 118.52 (C-6′′′), 117.45 (C-2′′), 116.31 (C-6), 114.36 (C-8), 114.21 (C-3′,5′), 112.48 (C-2′′′), 102.48 (C-4a), 58.69 (-OCH_3_), 56.43 (-OCH_3_), 20.43 (-CH_3_). HRMS (ESI)* m*/*z*: 509.18223 [M + Na]^+^, calcd. for C_28_H_22_NaO_8_ 509.12124.

*(2) General Method for the Synthesis of Compounds* ***2a–2d*** *(Describing One of the Parental Isoflavone Compounds as an Example)*

*Preparation of L-Aspartic Acid Dimethyl Ester*. L-aspartic acid dimethyl ester hydrochloride (0.20 g, 1.012 mmol) and K_2_CO_3_ (0.21 g, 1.518 mmol) were dissolved in 20 mL of deionized water. After stirring for 30 min and extraction using ethyl acetate, the ethyl acetate layer was collected. The water was removed from this layer using anhydrous Na_2_SO_4_. After the ethyl acetate layer was evaporated, the remaining ester was colorless.

*Preparation of Chloroacetyl Chloride Products of L-Aspartic Acid Dimethyl Ester*. A total of 0.2 g of L-aspartic acid dimethyl ester was dissolved in 20 mL of anhydrous ethyl acetate. Then, 2 eq of chloroacetyl chloride and 4 eq of anhydrous NaHCO_3_ were added at 0°C. The solution was stirred for 24 h at room temperature, and the filtrate was concentrated below 45°C after inorganic salt was removed by filtering.

The chloroacetyl chloride products of L-aspartic acid dimethyl ester were not fluorescent in the GF254 plate. Although these products can be spotted using the iodine-zinc method, the color is dark and is difficult to observe. Therefore, the chloroacetyl chloride products of L-aspartic acid dimethyl ester purified in a 300–400 mesh silica column (dichloromethane : methanol, 300 : 8) were directly pooled and used in the next step.

*Synthesis of Target Compounds* ***2a–2d***. The chloroacetyl chloride products of L-aspartic acid dimethyl ester (0.10 g, 0.621 mmol) as well as genistein (0.20 g, 0.740 mmol), K_2_CO_3_ (0.40 g, 2.894 mmol), and KI (0.12 g, 0.723 mmol) were dissolved in DMF (10.00 mL) and reacted for 4 h at 50~60°C. Then, 100 mL of water was added after the mixture was cooled. Afterwards, 10% hydrochloric acid was added to adjust the pH to 4, and the precipitate was extracted with ethyl acetate. The water was removed from the ethyl acetate layer using anhydrous Na_2_SO_4_, and the crude material was then purified in a 300–400 mesh silica column (dichloromethane : methanol, 20 : 1). The crude products of biochanin A and formononetin were then purified in 300–400 mesh silica columns (dichloromethane : methanol, 300 : 8).

*7-Acetyl-L-aspartic Acid Dimethyl Ester-O-genistein (* ***2a***). The product was obtained as a white solid (18.9%); m.p. 282.7~283.6°C. ^1^H-NMR (DMSO-*d*_6_) *δ* (ppm): 12.97 (s, 1H, 5-OH), 9.63 (s, 1H, 4′-OH), 8.70 (d,* J* = 8.1 Hz, 1H, -NH-), 8.45 (s, 1H, H-2), 7.40 (d,* J* = 9.7 Hz, 2H, H-2′,6′), 6.83 (d,* J* = 8.3 Hz, 2H, H-3′,5′), 6.65 (s, 1H, H-8), 6.44 (s, 1H, H-6), 4.76 (dt,* J* = 16.1, 7.9 Hz, 1H, -CH-), 4.72 (s, 2H, -OCH_2_-), 3.65 (s, 3H, -OCH_3_), 3.62 (s, 3H, -OCH_3_), 2.90~2.79 (m, 2H, CH_2_COOMe). ^13^C-NMR (DMSO-*d*_6_) *δ* (ppm): 180.95 (C-4), 171.28 (-COOCH_3_), 170.96 (-COOCH_3_), 167.47 (-COHN-), 163.90 (C-7), 162.16 (C-4′), 157.99 (C-5), 157.79 (C-8a), 155.03 (C-2), 130.65 (C-2′,6′), 123.09 (C-1′), 121.46 (C-3), 115.58 (C-3′,5′), 106.31 (C-4a), 99.18 (C-6), 93.73 (C-8), 67.39 (-OCH_2_-), 52.80 (-OCH_3_), 52.18 (-OCH_3_), 48.72 (-NHCH-), 35.84 (-COCH_2_-). HRMS (ESI)* m*/*z*: 470.10232 [M − H]^−^, calcd. for C_23_H_20_NO_10_ 470.10872.

*4*′*,7-Diacetyl-L-aspartic Acid Dimethyl Ester-O-genistein ( ****2b***). The product was obtained as a white solid (17.2%); m.p. 271.4~272.3°C. ^1^H-NMR (DMSO-*d*_6_) *δ* (ppm): 12.99 (s, 1H, 5-OH), 8.73 (d,* J* = 8.2 Hz, 2H, -NH-), 8.42 (s, 1H, H-2), 7.34 (d,* J* = 8.8 Hz, 2H, H-2′,6′), 6.89 (d,* J* = 8.4 Hz, 2H, H-3′,5′), 6.71 (s, 1H, H-8), 6.48 (s, 1H, H-6), 4.89 (dt,* J* = 16.3, 8.2 Hz, 2H, -CH-), 4.72 (s, 4H, -OCH_2_-), 3.91 (s, 6H, two -OCH_3_), 3.71 (s, 6H, two -OCH_3_), 2.92, 2.81 (m, 4H, CH_2_COOMe).^13^C-NMR (DMSO-*d*_6_) *δ* (ppm): 180.90 (C-4), 171.98, 171.22 (two -COOCH_3_), 168.81, 168.12 (two -COOCH_3_), 166.82, 165.89 (two -COHN-), 164.48 (C-7), 162.92 (C-4′), 157.48 (C-5), 156.21 (C-8a), 155.31 (C-2), 130.37 (C-2′,6′), 123.32 (C-1′), 121.58 (C-3), 114.38 (C-3′,5′), 106.78 (C-4a), 99.23 (C-6), 93.89 (C-8), 67.49, 66.64 (two -OCH_2_-), 56.68, 55.82 (two -OCH_3_), 53.88, 53.11 (two -OCH_3_), 48.65, 47.87 (two -NHCH-), 35.63, 33.81 (two -COCH_2_-). HRMS (ESI)* m*/*z*: 673.18836 [M + H]^+^, calcd. for C_31_H_33_N_2_O_15_ 673.18810. 

*7-Acetyl-L-aspartic Acid Dimethyl Ester-O-biochanin A (* ***2c***). The product was obtained as a white solid (31.8%); m.p. 191.1~191.9°C. ^1^H-NMR (DMSO-*d*_6_) *δ* (ppm): 12.93 (s, 1H, 5-OH), 8.76 (d,* J* = 8.2 Hz, 1H, -NH-), 8.52 (s, 1H, H-2), 7.58 (d,* J* = 8.6 Hz, 2H, H-2′,6′), 7.11 (d,* J* = 8.2 Hz, 2H, H-3′,5′), 6.81 (s, 1H, H-8), 6.63 (s, 1H, H-6), 4.95 (dt,* J* = 15.2, 7.6 Hz, 1H, -CH-), 4.76 (s, 2H, -OCH_2_-), 3.91 (s, 3H, -OCH_3_), 3.79 (s, 3H, -OCH_3_), 3.75 (s, 3H, -OCH_3_), 2.85~2.73 (m, 2H, CH_2_COOMe). ^13^C-NMR (DMSO-*d*_6_) *δ* (ppm): 180.88 (C-4), 171.49 (-COOCH_3_), 170.99 (-COOCH_3_), 166.37 (-COHN-), 163.82 (C-7), 162.38 (C-4′), 157.45 (C-5), 157.12 (C-8a), 155.19 (C-2), 130.78 (C-2′,6′), 123.61 (C-1′), 121.98 (C-3), 115.41 (C-3′,5′), 106.24 (C-4a), 99.51 (C-6), 93.22 (C-8), 66.48 (-OCH_2_-), 55.38 (-OCH_3_), 51.47 (-OCH_3_), 51.22 (-OCH_3_), 48.32 (-NHCH-), 35.19 (-COCH_2_-). HRMS (ESI)* m*/*z*: 486.14032 [M + H]^+^, calcd. for C_24_H_24_NO_10_ 486.14002. 

*7-Acetyl-L-aspartic Acid Dimethyl Ester-O-formononetin (* ***2d***). The product was obtained as a white solid (35.6%); m.p. 263.3~264.2°C. ^1^H-NMR (DMSO-*d*_6_) *δ* (ppm): 8.78 (d,* J* = 8.2 Hz, 1H, -NH-), 8.48 (s, 1H, H-2), 8.12 (d,* J* = 8.4 Hz, 1H, H-5), 7.63 (d,* J* = 9.7 Hz, 2H, H-2′,6′), 7.11 (d,* J* = 8.2 Hz, 2H, H-3′,5′), 6.98 (d,* J* = 8.3 Hz, 1H, H-6), 6.81 (s, 1H, H-8), 4.81 (dt,* J* = 15.4, 7.3 Hz, 1H, -CH-), 4.72 (s, 2H, -OCH_2_-), 3.83 (s, 3H, -OCH_3_), 3.71 (d,* J* = 15.4 Hz, 6H, two -OCH_3_), 2.75~2.63 (m, 2H, CH_2_COOMe). ^13^C-NMR (DMSO-*d*_6_) *δ* (ppm): 178.42 (C-4), 171.41 (-COOCH_3_), 170.28 (-COOCH_3_), 166.12 (-COHN-), 163.21 (C-7), 161.45 (C-4′), 159.12 (C-8a), 156.90 (C-2), 130.97 (C-2′,6′), 128.47 (C-5), 126.40 (C-1′), 125.32 (C-3), 118.35 (C-6), 116.46 (C-8), 115.14 (C-3′,5′), 103.48 (C-4a), 66.31 (-OCH_2_-), 56.72 (-OCH_3_), 51.95 (-OCH_3_), 51.17 (-OCH_3_), 47.22 (-NHCH-), 35.32 (-COCH_2_-). HRMS (ESI)* m*/*z*: 470.14429 [M + H]^+^, calcd. for C_24_H_24_NO_9_ 470.14511.

*(3) General Method for the Synthesis of Compounds* ***3a–3h*** *(Describing One of the Parental Isoflavone Compounds as an Example)*. Genistein (0.30 g, 1.110 mmol) and triethylamine (1.00 mL) were dissolved in anhydrous alcohol (25.00 mL), and a mixture of CCl_4_ (5.00 mL) and either diisopropyl phosphite (0.18 g, 1.083 mmol) or diethyl phosphite (0.18 g, 1.304 mmol) was then added dropwise to the mixture of genistein and triethylamine at 5°C for 2 h. The final mixture was gradually brought to room temperature and reacted for 24 h. After the reaction, the mixture was concentrated under low pressure. The residue was dissolved with ethyl acetate and then filtered to remove insoluble matter. The crude material was then purified in a 300–400 mesh silica column (dichloromethane : methanol, 12 : 1). The crude products of biochanin A, formononetin, the second reaction of genistein, the second reaction of biochanin A, and the second reaction of formononetin were then individually purified in 300–400 mesh silica columns (dichloromethane : methanol, 300 : 8). 

*7-Diethyl Phosphite-O-genistein (* ***3a***). The product was obtained as a white solid (15.1%); m.p. 283.1~283.8°C.^1^H-NMR (DMSO-*d*_*6*_) *δ* (ppm): 13.01 (s, 1H, 5-OH), 9.64 (s, 1H, 4′-OH), 8.50 (s, 1H, H-2), 7.41 (d,* J* = 8.5 Hz, 2H, H-2′,6′), 6.95 (d,* J* = 2.0 Hz, 1H, H-8), 6.84 (d,* J* = 8.6 Hz, 2H, H-3′,5′), 6.70 (d,* J* = 2.1 Hz, 1H, H-6), 4.25–4.18 (m, 4H, two -OCH_2_-), 1.27 (q,* J* = 6.8 Hz, 6H, two -CH_3_).^13^C-NMR (DMSO-*d*_*6*_) *δ* (ppm): 181.24 (C-4), 162.22 (C-7), 158.10 (C-4′), 157.17 (C-5), 156.09 (C-8a), 155.59 (C-2), 130.67 (C-2′,6′), 123.45 (C-1′), 121.18 (C-3), 115.60 (C-3′,5′), 108.56 (C-4a), 103.34 (C-6), 99.19 (C-8), 65.35 (two -OCH_2_-), 17.35 (two -CH_3_). HRMS (ESI)* m*/*z*: 405.08931 [M − H]^−^, calcd. for C_19_H_18_O_8_P 405.07393.

*4*′*-Diethyl Phosphite-O-genistein ( ****3b***). The product was obtained as a white solid (13.2%); m.p. 278.2~278.9°C. ^1^H-NMR (DMSO-*d*_*6*_) *δ* (ppm): 13.02 (s, 1H, 5-OH), 10.92 (s, 1H, 7-OH), 8.51 (s, 1H, H-2), 7.40 (d,* J* = 7.9 Hz, 2H, H-2′,6′), 6.98 (d,* J* = 2.0 Hz, 1H, H-8), 6.84 (t,* J* = 8.1 Hz, 2H, H-3′,5′), 6.71 (d,* J* = 2.1 Hz, 1H, H-6), 4.26–4.17 (m, 4H, two -OCH_2_-), 1.29 (q,* J* = 6.8 Hz, 6H, two -CH_3_). ^13^C-NMR (DMSO-*d*_*6*_) *δ* (ppm): 181.26 (C-4), 162.26 (C-7), 158.11 (C-4′), 157.18 (C-5), 155.89 (C-8a), 155.62 (C-2), 130.67 (C-2′,6′), 123.46 (C-1′), 121.16 (C-3), 115.61 (C-3′,5′), 108.71 (C-4a), 103.38 (C-6), 99.30 (C-8), 65.36 (two -OCH_2_-), 16.36 (two -CH_3_). HRMS (ESI)* m*/*z*: 405.09234 [M − H]^−^, calcd. for C_19_H_18_O_8_P 405.07393.

*7-Diethyl Phosphite-O-biochanin A (* ***3c***). The product was obtained as a white solid (35.3%); m.p. 181.6~182.7°C. ^1^H-NMR (DMSO-*d*_*6*_) *δ* (ppm): 12.99 (s, 1H, 5-OH), 8.55 (s, 1H, H-2), 7.53 (d,* J* = 8.7 Hz, 2H, H-2′,6′), 7.02 (t,* J* = 9.0 Hz, 2H, H-3′,5′), 6.99 (d,* J* = 2.0 Hz, 1H, H-8), 6.72 (d,* J* = 2.1 Hz, 1H, H-6), 4.27–4.17 (m, 4H, two -OCH_2_-), 3.80 (d,* J* = 4.2 Hz, 3H, -OCH_3_), 1.30 (t,* J* = 7.0 Hz, 6H, two -CH_3_). ^13^C-NMR (DMSO-*d*_*6*_) *δ* (ppm): 181.17 (C-4), 162.25 (C-7), 159.82 (C-4′), 157.20 (C-5), 156.92 (C-8a), 155.89 (C-2), 130.68 (C-2′,6′), 123.14 (C-1′), 122.89 (C-3), 114.26 (C-3′,5′), 108.71 (C-4a), 103.48 (C-6), 99.38 (C-8), 65.37 (two -OCH_2_-), 55.67 (-OCH3), 16.36 (two -CH_3_). HRMS (ESI)* m*/*z*: 421.10539 [M + H]^+^, calcd. for C_20_H_22_O_8_P 421.10523.

*7-Diethyl Phosphite-O-formononetin (* ***3d***). The product was obtained as a white solid (32.1%); m.p. 271.1~271.8°C. ^1^H-NMR (DMSO-*d*_*6*_) *δ* (ppm): 8.47 (s, 1H, H-2), 8.18 (d,* J* = 8.8 Hz, 1H, H-5), 7.56–7.41 (m, 3H, H-2′,6′ and H-8), 7.38 (dd,* J* = 8.8, 2.1 Hz, 1H, H-6), 7.00 (d,* J* = 8.8 Hz, 2H, H-3′,5′), 4.25–4.16 (m, 4H, two -OCH_2_-), 3.78 (s, 3H, -OCH_3_), 1.28 (t,* J* = 7.0 Hz, 5H, two -CH_3_). ^13^C-NMR (DMSO-*d*_*6*_) *δ* (ppm): 175.26 (C-4), 159.67 (C-7), 156.77 (C-4′), 154.66 (C-2), 154.57 (C-8a), 130.60 (C-2′,6′), 128.32 (C-5), 124.19 (C-1′), 124.04 (C-3), 121.46 (C-6), 118.65 (C-8), 114.16 (C-3′,5′), 97.60 (C-4a), 65.53 (two -OCH_2_-), 55.60 (-OCH_3_), 16.34 (two -CH_3_). HRMS (ESI)* m*/*z*: 405.12301 [M + H]^+^, calcd. for C_20_H_21_O_7_P 405.11032.

*7-Diisopropyl Phosphite-O-genistein (* ***3e***). The product was obtained as a white solid (16.3%); m.p. 282.2~282.9°C. ^1^H-NMR (DMSO-*d*_*6*_) *δ* (ppm): 13.01 (s, 1H, 5-OH), 9.65 (s, 1H, 4′-OH), 8.51 (s, 1H, H-2), 7.41 (d,* J* = 8.5 Hz, 2H, H-2′,6′), 6.96 (d,* J* = 2.0 Hz, 1H, H-8), 6.84 (d,* J* = 8.6 Hz, 2H, H-3′,5′), 6.70 (d,* J* = 2.0 Hz, 1H, H-6), 4.72 (dq,* J* = 12.6, 6.3 Hz, 2H, two -CH-), 1.31 (dd,* J* = 11.8, 6.2 Hz, 12H, four -CH_3_). ^13^C-NMR (DMSO-*d*_*6*_) *δ* (ppm): 181.25 (C-4), 162.22 (C-7), 158.10 (C-4′), 157.17 (C-5), 156.09 (C-8a), 155.61 (C-2), 130.68 (C-2′,6′), 123.45 (C-1′), 121.18 (C-3), 115.61 (C-3′,5′), 108.56 (C-4a), 103.35 (C-6), 99.20 (C-8), 74.37 (two -CH-), 23.73 (four -CH_3_). HRMS (ESI)* m*/*z*: 433.11374 [M − H]^−^, calcd. for C_21_H_22_O_8_P 433.10523.

*4*′*-Diisopropyl Phosphite-O-genistein ( ****3f***). The product was obtained as a white solid (22.9%); m.p. 273.8~274.6°C. ^1^H-NMR (DMSO-*d*_*6*_) *δ* (ppm): 12.96 (s, 1H, 5-OH), 10.92 (s, 1H, 7-OH), 8.58 (s, 1H, H-2), 7.54 (d,* J* = 8.7 Hz, 2H, H-2′,6′), 7.15 (d,* J* = 2.1 Hz, 1H, H-8), 7.04 (d,* J* = 8.7 Hz, 2H, H-3′,5′), 6.82 (d,* J* = 2.1 Hz, 1H, H-6), 4.82 (m, 2H, two -CH-), 1.33 (dd,* J* = 11.7, 6.2 Hz, 12H, four -CH_3_). ^13^C-NMR (DMSO-*d*_*6*_) *δ* (ppm): 181.35 (C-4), 161.84 (C-7), 159.80 (C-4′), 156.04 (C-5), 152.23 (C-8a), 151.47 (C-2), 130.70 (C-2′,6′), 125.05 (C-1′), 123.15 (C-3), 114.27 (C-3′,5′), 109.94 (C-4a), 105.23 (C-6), 101.35 (C-8), 76.40 (two -CH-), 23.50 (four -CH_3_). HRMS (ESI)* m*/*z*: 433.11492 [M − H]^−^, calcd. for C_21_H_22_O_8_P 433.10523.

*7-Diisopropyl Phosphite-O-biochanin A (* ***3g***). The product was obtained as a white solid (31.3%); m.p. 191.1~191.8°C. ^1^H-NMR (DMSO-*d*_*6*_) *δ* (ppm): 12.98 (s, 1H, 5-OH), 8.55 (s, 1H, H-2), 7.52 (dd,* J* = 8.2, 5.4 Hz, 2H, H-2′,6′), 7.03 (d,* J* = 8.7 Hz, 2H, H-3′,5′), 6.97 (d,* J* = 2.1 Hz, 1H, H-8), 6.71 (d,* J* = 2.1 Hz, 1H, H-6), 4.77–4.68 (m, 2H, two -CH-), 3.80 (s, 3H, -OCH_3_), 1.31 (dd,* J* = 11.7, 6.2 Hz, 12H, four -CH_3_). ^13^C-NMR (DMSO-*d*_*6*_) *δ* (ppm): 181.16 (C-4), 162.21 (C-7), 159.81 (C-4′), 157.18 (C-5), 156.14 (C-8a), 155.91 (C-2), 130.68 (C-2′,6′), 123.13 (C-1′), 122.90 (C-3), 114.26 (C-3′,5′), 108.57 (C-4a), 103.41 (C-6), 99.24 (C-8), 74.38 (two -CH-), 55.67 (-OCH_3_), 23.73 (four -CH_3_). HRMS (ESI)* m*/*z*: 449.13647 [M + H]^+^, calcd. for C_22_H_26_O_8_P 449.13653.

*7-Diisopropyl Phosphite-O-formononetin (* ***3h***). The product was obtained as a white solid (34.2%); m.p. 263.2~263.9°C. ^1^H-NMR (DMSO-*d*_*6*_) *δ* (ppm): 8.50 (s, 1H, H-2), 8.19 (d,* J* = 8.8 Hz, 1H, H-5), 7.54 (t,* J* = 5.7 Hz, 2H, H-2′,6′), 7.50 (d,* J* = 2.0 Hz, 1H, H-8), 7.38 (dd,* J* = 8.8, 2.2 Hz, 1H, H-6), 7.01 (t,* J* = 7.8 Hz, 2H, H-3′,5′), 4.73 (dq,* J* = 12.5, 6.3 Hz, 2H, two -CH-), 3.80 (s, 3H, -OCH_3_), 1.31 (dd,* J* = 15.5, 6.2 Hz, 12H, four -CH_3_). ^13^C-NMR (DMSO-*d*_*6*_) *δ* (ppm): 175.10 (C-4), 159.61 (C-7), 156.77 (C-4′), 154.83 (C-8a), 154.60 (C-2), 130.57 (C-2′,6′), 128.23 (C-5), 124.16 (C-1′), 121.36 (C-3), 118.54 (C-6 and C-8), 114.15 (C-3′,5′), 109.21 (C-4a), 74.33 (two -CH-), 55.63 (-OCH_3_), 23.73 (four -CH_3_). HRMS (ESI)* m*/*z*: 433.15821 [M + H]^+^, calcd. for C_22_H_26_O_7_P 433.14162.

*(4) General Method for the Synthesis of Compounds* ***4a–4j*** *(Describing One of the Parental Isoflavone Compounds as an Example)*. Genistein (0.20 g, 0.740 mmol) and 1,2-dibromoethane (0.68 mL) or hexamethylene dibromide (0.68 mL) and anhydrous K_2_CO_3_ (0.20 g, 1.447 mmol) were dissolved in anhydrous alcohol (10.00 mL). The mixture was stirred under reflux for 24 h, and the insoluble matter was then removed by filtering. The filtrate was concentrated under low pressure. The crude material was then purified in a 300–400 mesh silica column (petroleum ether : ethyl acetate, 6 : 1). The crude product of biochanin A was then purified in a 300–400 mesh silica column (petroleum ether : ethyl acetate : methanol, 6 : 2 : 1). The crude products of formononetin and the second reaction of genistein were purified in 300*–*400 mesh silica columns (petroleum ether : ethyl acetate, 6 : 1). The crude products of the second reaction of biochanin A and the second reaction of formononetin were purified in 300–400 mesh silica columns (petroleum ether : ethyl acetate, 3 : 1).

*7-2-Bromo-ethoxy-genistein (* ***4a***). The product was obtained as a white solid (25.9%); m.p. 274.2~274.9°C. ^1^H-NMR (DMSO-*d*_*6*_) *δ* (ppm): 12.94 (s, 1H, 5-OH), 9.77 (s, 1H, 4′-OH), 8.41 (s, 1H, H-2), 7.51 (d,* J* = 8.4 Hz, 2H, H-2′,6′), 6.95 (t,* J* = 9.1 Hz, 2H, H-3′,5′), 6.67 (s, 1H, H-8), 6.48 (s, 1H, H-6), 4.21 (t,* J* = 6.3 Hz, 2H, -OCH_2_-), 3.41 (t,* J* = 7.2 Hz, 2H, BrCH_2_-). ^13^C-NMR (DMSO-*d*_*6*_) *δ* (ppm): 175.04 (C-4), 161.95 (C-7), 161.35 (C-4′), 159.38 (C-5), 158.05 (C-8a), 153.52 (C-2), 130.54 (C-2′,6′), 124.79 (C-1′), 123.54 (C-3), 114.05 (C-3′,5′), 105.55 (C-4a), 98.54 (C-6), 92.52 (C-8), 68.61 (-OCH_2_-), 34.89 (BrCH_2_-). HRMS (ESI)* m*/*z*: 374.97623 [M − H]^−^, calcd. for C_17_H_12_BrO_5_ 374.98681.

*4*′*,7-di-2-Bromo-ethoxy-genistein ( ****4b***). The product was obtained as a white solid (28.2%); m.p. 261.4~262.2°C. ^1^H-NMR (DMSO-*d*_*6*_) *δ* (ppm): 12.92 (s, 1H, 5-OH), 8.51 (s, 1H, H-2), 7.47 (d,* J *= 8.2 Hz, 2H, H-2′,6′), 7.17 (d,* J* = 8.3 Hz, 2H, H-3′,5′), 6.71 (s, 1H, H-8), 6.46 (s, 1H, H-6), 4.26 (t,* J* = 7.2 Hz, 4H, two -OCH_2_-), 3.57 (t,* J* = 6.2 Hz, 4H, two BrCH_2_-). ^13^C-NMR (DMSO-*d*_*6*_) *δ* (ppm): 180.78 (C-4), 164.30 (C-7), 162.29 (C-4′), 158.35 (C-5), 157.95 (C-8a), 155.42 (C-2), 130.75 (C-2′,6′), 123.73 (C-1′), 122.60 (C-3), 114.93 (C-3′,5′), 106.16 (C-4a), 98.98 (C-6), 93.61 (C-8), 69.02 (-OCH_2_-), 68.27 (-OCH_2_-), 35.72 (BrCH_2_-), 33.72 (BrCH_2_-). HRMS (ESI)* m*/*z*: 482.94451 [M + H]^+^, calcd. for C_19_H_17_Br_2_O_5_ 482.94427.

*4*′*-2-Bromo-ethoxy-genistein ( ****4c***). The product was obtained as a white solid (15.2%); m.p. 278.6~279.4°C. ^1^H-NMR (DMSO-*d*_*6*_) *δ* (ppm): 13.07 (s, 1H, 5-OH), 11.07 (s, 1H, 7-OH), 8.21 (s, 1H, H-2), 7.32 (d,* J* = 8.2 Hz, 2H, H-2′,6′), 6.77 (d,* J* = 8.1 Hz, 2H, H-3′,5′), 6.48 (s, 1H, H-8), 6.24 (s, 1H, H-6), 4.20 (t,* J* = 7.2 Hz, 2H, -OCH_2_-), 3.58 (t,* J* = 7.2 Hz, 2H, BrCH_2_-). ^13^C-NMR (DMSO-*d*_*6*_) *δ* (ppm): 181.45 (C-4), 160.58 (C-7), 159.68 (C-4′), 156.56 (C-5), 155.63 (C-8a), 153.76 (C-2), 130.25 (C-2′,6′), 124.43 (C-1′), 124.04 (C-3), 114.51 (C-3′,5′), 104.93 (C-4a), 97.63 (C-6), 93.10 (C-8), 62.94 (-OCH_2_-), 35.14 (BrCH_2_-). HRMS (ESI)* m*/*z*: 374.97252 [M − H]^−^, calcd. for C_17_H_12_BrO_5_ 374.98681.

*7-2-Bromo-ethoxy-biochanin A (* ***4d***). The product was obtained as a white solid (38.3%); m.p. 193.7~194.5°C. ^1^H-NMR (DMSO-*d*_*6*_) *δ* (ppm): 13.15 (s, 1H, 5-OH), 8.47 (s, 1H, H-2), 7.54 (d,* J* = 8.3 Hz, 2H, H-2′,6′), 7.21 (d,* J* = 8.1 Hz, 2H, H-3′,5′), 6.47 (s, 1H, H-8), 6.38 (s, 1H, H-6), 4.48 (t,* J* = 6.2 Hz, 2H, -OCH_2_-), 3.82 (s, 3H, -OCH_3_), 3.64 (t,* J* = 7.1 Hz, 2H, BrCH_2_-). ^13^C-NMR (DMSO-*d*_*6*_) *δ* (ppm): 181.24 (C-4), 164.56 (C-7), 160.58 (C-4′), 159.68 (C-5), 158.19 (C-8a), 154.12 (C-2), 131.62 (C-2′,6′), 123.07 (C-1′), 121.26 (C-3), 114.63 (C-3′,5′), 104.93 (C-4a), 100.33 (C-6), 94.73 (C-8), 62.94 (-OCH_2_-), 56.37 (-OCH_3_), 30.51 (BrCH_2_-). HRMS (ESI)* m*/*z*: 391.01821 [M + H]^+^, calcd. for C_18_H_16_BrO_5_ 391.01811.

*7-2-Bromo-ethoxy-formononetin (* ***4e***). The product was obtained as a white solid (41.2%); m.p. 248.5~249.1°C. ^1^H-NMR (DMSO-*d*_*6*_) *δ* (ppm): 8.46 (s, 1H, H-2), 8.05 (d,* J* = 8.7 Hz, 1H, H-5), 7.53 (d,* J* = 8.2 Hz, 2H, H-2′,6′), 7.23 (s, 1H, H-8), 7.12 (d,* J* = 8.3 Hz, 1H, H-6), 7.01 (d,* J* = 8.1 Hz, 2H, H-3′,5′), 4.23 (t,* J* = 6.3 Hz, 2H, -OCH_2_-), 3.80 (s, -OCH_3_, 3H), 3.55 (t,* J* = 7.2 Hz, 2H, BrCH_2_-). ^13^C-NMR (DMSO-*d*_*6*_) *δ* (ppm): 175.09 (C-4), 162.72 (C-7), 159.49 (C-4′), 157.81 (C-8a), 154.07 (C-2), 130.56 (C-2′,6′), 127.58 (C-5), 124.48 (C-1′), 123.87 (C-3), 118.38 (C-6), 115.49 (s, C-8), 114.10 (C-3′,5′), 101.90 (C-4a), 65.50 (-OCH_2_-), 55.62 (-OCH_3_), 35.35 (BrCH_2_-). HRMS (ESI)* m*/*z*: 375.03438 [M + H]^+^, calcd. for C_18_H_16_BrO_4_ 375.02320.

*7-6-Bromo-hexyloxy-genistein (* ***4f***). The product was obtained as a white solid (21.1%); m.p. 252.7~253.5°C. ^1^H-NMR (DMSO-*d*_*6*_) *δ* (ppm): 12.95 (s, 1H, 5-OH), 9.63 (s, 1H, 4′-OH), 8.17 (s, 1H, H-2), 7.42 (d,* J* = 8.5 Hz, 2H, H-2′,6′), 6.96 (d,* J* = 8.2 Hz, 2H, H-3′,5′), 6.63 (s, 1H, H-8), 6.48 (s, 1H, H-6), 4.09 (t,* J* = 6.1 Hz, 2H, -OCH_2_-), 3.54 (t,* J* = 7.1 Hz, 2H, BrCH_2_-), 1.83 (m,* J* = 13.2, 6.1 Hz, 2H, -CH_2_-), 1.65 (m,* J* = 13.2, 6.1 Hz, 2H, -CH_2_-), 1.59 (m,* J* = 14.3, 7.3 Hz, 2H, -CH_2_-), 1.43 (m,* J* = 17.3, 11.4 Hz, 2H, -CH_2_-). ^13^C-NMR (DMSO-*d*_*6*_) *δ* (ppm): 174.13 (C-4), 163.37 (C-7), 160.72 (C-4′), 159.67 (C-5), 158.72 (C-8a), 151.29 (C-2), 130.75 (C-2′,6′), 125.10 (C-1′), 124.75 (C-3), 114.44 (C-3′,5′), 109.45 (C-4a), 97.51 (C-6), 93.73 (C-8), 67.80 (-OCH_2_-), 35.60 (BrCH_2_-), 32.67 (-CH_2_-), 28.98 (-CH_2_-), 27.78 (-CH_2_-), 25.16 (-CH_2_-). HRMS (ESI)* m*/*z*: 431.04228 [M − H]^−^, calcd. for C_21_H_20_BrO_5_ 431.04941.

*4*′*,7-di-6-Bromo-hexyloxy-genistein ( ****4g***). The product was obtained as a white solid (18.1%); m.p. 244.1~244.9°C. ^1^H-NMR (DMSO-*d*_*6*_) *δ* (ppm): 12.92 (s, 1H, 5-OH), 8.37 (s, 1H, H-2), 7.44 (d,* J* = 8.1 Hz, 2H, H-2′,6′), 7.09 (d,* J* = 8.2 Hz, 2H, H-3′,5′), 6.74 (s, 1H, H-8), 6.58 (s, 1H, H-6), 4.02 (t,* J* = 6.9 Hz, 4H, -OCH_2_-), 3.45 (t,* J* = 7.3 Hz, 4H, BrCH_2_-), 1.89 (m,* J* = 13.5, 6.6 Hz, 4H, -CH_2_-), 1.65 (m,* J* = 13.2, 6.4 Hz, 4H, -CH_2_-), 1.56 (m,* J* = 14.2, 7.3 Hz, 4H, -CH_2_-), 1.43 (m,* J* = 17.3, 11.4 Hz, 4H, -CH_2_-). ^13^C-NMR (DMSO-*d*_*6*_) *δ* (ppm): 174.12 (C-4), 163.37 (C-7), 160.71 (C-4′), 159.67 (C-5), 159.31 (C-8a), 151.31 (C-2), 130.76 (C-2′,6′), 125.08 (C-1′), 124.87 (C-3), 113.91 (C-3′,5′), 109.44 (C-4a), 97.50 (C-6), 93.73 (C-8), 68.62 (-OCH_2_-), 65.66 (-OCH_2_-), 32.67 (two BrCH_2_-), 28.71 (two -CH_2_-), 27.60 (two -CH_2_-), 24.99 (two -CH_2_-), 22.27 (two -CH_2_-). HRMS (ESI)* m*/*z*: 595.06991 [M + H]^+^, calcd. for C_27_H_33_Br_2_O_5_ 595.06947.

*4*′*-6-Bromo-hexyloxy-genistein ( ****4h***). The product was obtained as a white solid (22.4%); m.p. 267.2~267.9°C. ^1^H-NMR (DMSO-*d*_*6*_) *δ* (ppm): 12.96 (s, 1H, 5-OH), 11.02 (s, 1H, 7-OH), 8.41 (s, 1H, H-2), 7.57 (d,* J* = 8.3 Hz, 2H, H-2′,6′), 7.19 (t,* J* = 8.2 Hz, 2H, H-3′,5′), 6.76 (s, 1H, H-8), 6.63 (s, 1H, H-6), 4.21 (t,* J* = 6.3 Hz, 2H, -OCH_2_-), 3.61 (t,* J* =7.3 Hz, 2H, BrCH_2_-), 1.78 (m,* J* = 13.1, 6.3 Hz, 2H, -CH_2_-), 1.57 (m,* J* = 13.1, 6.3 Hz, 2H, -CH_2_-), 1.47 (m,* J* = 13.8, 7.1 Hz, 2H, -CH_2_-), 1.38 (m,* J* = 17.1, 11.6 Hz, 2H, -CH_2_-). ^13^C-NMR (DMSO-*d*_*6*_) *δ* (ppm): 180.77 (C-4), 165.16 (C-7), 162.22 (C-4′), 159.68 (C-5), 157.99 (C-8a), 155.17 (C-2), 130.64 (C-2′,6′), 123.25 (C-1′), 122.64 (C-3), 114.21 (C-3′,5′), 105.81 (C-4a), 98.87 (C-6), 93.34 (C-8), 68.87 (-OCH_2_-), 35.59 (BrCH_2_-), 32.62 (-CH_2_-), 27.71 (-CH_2_-), 25.02 (-CH_2_-), 21.20 (-CH_2_-). HRMS (ESI)* m*/*z*: 431.05478 [M − H]^−^, calcd. for C_21_H_20_BrO_5_ 431.04941.

*7-6-Bromo-hexyloxy-biochanin A (* ***4i***). The product was obtained as a white solid (43.8%); m.p. 184.2~184.9°C. ^1^H-NMR (DMSO-*d*_*6*_) *δ* (ppm): 12.92 (s, 1H, 5-OH), 8.46 (s, 1H, H-2), 7.52 (d,* J* = 8.2 Hz, 2H, H-2′,6′), 7.02 (d,* J* = 8.3 Hz, 2H, H-3′,5′), 6.66 (s, 1H, H-8), 6.41 (s, 1H, H-6), 4.09 (t,* J* = 6.3 Hz, 2H, -OCH_2_-), 3.80 (s, 3H, -OCH_3_), 3.40 (t,* J* = 7.1 Hz, 2H, BrCH_2_-), 1.73 (m,* J* = 13.7, 6.7 Hz, 2H, -CH_2_-), 1.52 (m,* J* = 13.4, 6.8 Hz, 2H, -CH_2_-), 1.44 (m,* J* = 14.7, 7.1 Hz, 2H, -CH_2_-), 1.37 (m,* J* = 17.9, 11.2 Hz, 2H, -CH_2_-). ^13^C-NMR (DMSO-*d*_*6*_) *δ* (ppm): 180.77 (C-4), 165.19 (C-7), 162.22 (C-4′), 159.69 (C-5), 158.00 (C-8a), 155.18 (C-2), 130.65 (C-2′,6′), 123.26 (C-1′), 122.63 (C-3), 114.21 (C-3′,5′), 105.80 (C-4a), 98.88 (C-6), 93.35 (C-8), 65.66 (-OCH_2_-), 55.66 (s, -OCH_3_), 29.69 (BrCH_2_-), 28.84 (-CH_2_-), 25.94 (-CH_2_-), 25.74 (-CH_2_-), 22.63 (-CH_2_-). HRMS (ESI)* m*/*z*: 447.08082 [M + H]^+^, calcd. for C_22_H_24_BrO_5_ 447.08071.

*7-6-Bromo-hexyloxy-formononetin (* ***4j***). The product was obtained as a white solid (48.9%); m.p. 246.1~246.8°C. ^1^H-NMR (DMSO-*d*_*6*_) *δ* (ppm): 8.42 (s, 1H, H-2), 8.03 (d,* J* = 8.8 Hz, 1H, H-5), 7.53 (d,* J* = 8.1 Hz, 2H, H-2′,6′), 7.16 (s, 1H, H-8), 7.00 (d,* J* = 8.2 Hz, 2H, H-3′,5′), 6.88 (d,* J* = 8.2 Hz, 1H, H-6), 4.13 (t,* J* = 7.8 Hz, 2H, -OCH_2_-), 3.80 (s, -OCH_3_, 3H), 3.55 (t,* J* = 7.2 Hz, 2H, BrCH_2_-), 2.01 (m,* J* = 13.4, 6.2 Hz, 2H, -CH_2_-), 1.83 (m,* J* = 13.1, 6.1 Hz, 2H, -CH_2_-), 1.72 (m,* J* = 13.2, 6.1 Hz, 2H, -CH_2_-), 1.65 (m,* J* = 13.2, 6.7 Hz, 2H, -CH_2_-). ^13^C-NMR (DMSO-*d*_*6*_) *δ* (ppm): 175.11 (C-4), 163.57 (C-7), 159.48 (C-4′), 157.92 (C-8a), 153.93 (C-2), 130.54 (C-2′,6′), 127.41 (C-5), 124.55 (C-1′), 123.83 (C-3), 117.97 (C-6), 115.50 (C-8), 114.09 (C-3′,5′), 101.47 (C-4a), 64.17 (-OCH_2_-), 55.62 (-OCH_3_), 35.59 (BrCH_2_-), 32.62 (-CH_2_-), 28.46 (-CH_2_-), 25.49 (-CH_2_-), 21.19 (-CH_2_-). HRMS (ESI)* m*/*z*: 431.05480 [M + H]^+^, calcd. for C_22_H_24_BrO_4_ 431.08580.

*(5) General Method for the Synthesis of Compounds* ***5a–5d*** *(Describing One of the Parental Isoflavone Compounds as an Example)*. Genistein (0.50 g, 1.850 mmol), cinnamic acid (0.27 g, 1.820 mmol), DCC (0.560, 2.710 mmol), and pyridine (1.00 mL) were dissolved in THF (20.00 mL). The mixture was reacted at 80°C for 4 h. The crude material was then purified in a 300–400 mesh silica column (petroleum ether : ethyl acetate : methanol, 6 : 2 : 1). The crude product of biochanin A was purified in a 300–400 mesh silica column (petroleum ether : ethyl acetate : methanol, 6 : 2 : 1), and the crude product of formononetin was then purified in a 300–400 mesh silica column (dichloromethane : methanol, 300 : 8).

*7-Cinnamic Acid-O-genistein (* ***5a***). The product was obtained as a white solid (17.8%); m.p. 270.2~271.1°C. ^1^H-NMR (DMSO-*d*_*6*_) *δ* (ppm): 13.00 (s, 1H, 5-OH), 9.65 (s, 1H, 4′-OH), 8.53 (s, 1H, H-2), 7.96–7.90 (d,* J* = 15.6 Hz, 1H, H-3′′), 7.85–7.82 (m, 2H, H-2′′′,6′′′), 7.49 (dd,* J* = 5.2, 1.7 Hz, 3H, H-3′′′,5′′′, H-4′′′), 7.43 (d,* J* = 8.5 Hz, 2H, H-2′,6′), 7.11 (d,* J* = 2.0 Hz, 1H, H-8), 6.94–6.89 (d,* J* = 15.5 Hz, 1H, H-2′′), 6.86 (d,* J* = 8.5 Hz, 2H, H-3′,5′), 6.78 (d,* J* = 2.0 Hz, 1H, H-6). ^13^C-NMR (DMSO-*d*_*6*_) *δ* (ppm): 181.44 (C-4), 164.55 (C-1′′), 161.83 (C-7), 158.10 (C-4′), 157.08 (C-5), 156.92 (C-8a), 156.30 (C-2), 147.79 (C-3′′), 134.22 (C-1′′′), 131.59 (C-4′′′), 130.69 (C-2′,6′), 129.53 (C-3′′′,5′′′), 129.26 (C-2′′′,6′′′), 123.45 (C-1′), 121.26 (C-3), 117.11 (C-2′′), 115.61 (C-3′,5′), 109.29 (C-4a), 105.77 (C-6), 101.87 (C-8). HRMS (ESI)* m*/*z*: 399.07534 [M − H]^−^, calcd. for C_24_H_15_O_6_ 399.08686.

*4*′*-Cinnamic Acid-O-genistein ( ****5b***). The product was obtained as a white solid (25.5%); m.p. 278.1~278.9°C. ^1^H-NMR (DMSO-*d*_*6*_) *δ* (ppm): 13.01 (s, 1H, 5-OH), 10.82 (s, 1H, 7-OH), 8.41 (s, 1H, H-2), 7.82–7.80 (d,* J* = 15.6 Hz, 1H, H-3′′), 7.74–7.69 (m, 2H, H-2′′′,6′′′), 7.31 (dd,* J* = 5.2, 1.7 Hz, 3H, H-3′′′,5′′′, H-4′′′), 7.24 (d,* J* = 8.5 Hz, 2H, H-2′,6′), 6.81 (d,* J* = 2.0 Hz, 1H, H-8), 6.64–6.61 (d,* J* = 15.5 Hz, 1H, H-2′′), 6.56 (d,* J* = 8.5 Hz, 2H, H-3′,5′), 6.42 (d,* J* = 2.0 Hz, 1H, H-6). ^13^C-NMR (DMSO-*d*_*6*_) *δ* (ppm): 180.80 (C-4), 164.88 (C-1′′), 162.17 (C-7), 158.40 (C-4′), 156.49 (C-5), 156.27 (C-8a), 155.97 (C-2), 147.71 (C-3′′), 136.11 (C-1′′′), 131.28 (C-4′′′), 130.29 (C-2′,6′), 129.50 (C-3′′′,5′′′), 128.11 (C-2′′′,6′′′), 123.41 (C-1′), 121.53 (C-3), 117.22 (C-2′′), 115.25 (C-3′,5′), 109.16 (C-4a), 105.56 (C-6), 101.47 (C-8). HRMS (ESI)* m*/*z*: 399.09821 [M − H]^−^, calcd. for C_24_H_15_O_6_ 399.08686.

*7-Cinnamic Acid-O-biochanin A (* ***5c***). The product was obtained as a white solid (41.3%); m.p. 176.5~177.3°C. ^1^H-NMR (DMSO-*d*_*6*_) *δ* (ppm): 13.04 (s, 1H, 5-OH), 8.60 (s, 1H, H-2), 7.93–7.86 (d,* J* = 15.1 Hz, 1H, H-3′′), 7.83–7.77 (m, 2H, H-2′′′,6′′′), 7.54 (dd,* J* = 5.1, 1.9 Hz, 3H, H-3′′′,5′′′, H-4′′′), 7.44 (d,* J* = 8.6 Hz, 2H, H-2′,6′), 6.95 (d,* J* = 2.0 Hz, 1H, H-8), 6.79–6.75 (d,* J* = 15.8 Hz, 1H, H-2′′), 6.71 (d,* J* = 8.4 Hz, 2H, H-3′,5′), 6.66 (d,* J* = 2.0 Hz, 1H, H-6). 3.84 (s, 3H, -OCH_3_). ^13^C-NMR (DMSO-*d*_*6*_) *δ*(ppm): 181.35 (C-5), 164.14 (C-1′′), 161.37 (C-7), 158.28 (C-4′), 157.38 (C-5), 156.86 (C-8a), 156.12 (C-2), 147.24 (C-3′′), 133.38 (C-1′′′), 131.22 (C-4′′′), 130.17 (C-2′,6′), 128.48 (C-3′′′,5′′′), 128.09 (C-2′′′,6′′′), 123.20 (C-1′), 121.47 (C-3), 117.23 (C-2′′), 115.98 (C-3′,5′), 108.66 (C-4a), 105.34 (C-6), 101.35 (C-8), 55.68 (-OCH_3_). HRMS (ESI)* m*/*z*: 415.11832 [M + H]^+^, calcd. for C_25_H_19_O_6_ 415.11817.

*7-Cinnamic Acid-O-formononetin (* ***5d***). The product was obtained as a white solid (38.9%); m.p. 246.1~247.1°C. ^1^H-NMR (DMSO-*d*_6_) *δ* (ppm): 8.48 (s, 1H, H-2), 8.03 (d,* J* = 8.3 Hz, 1H, H-5), 7.85 (d,* J* = 16.2 Hz, 1H, H-3′′), 7.61 (m, 2H, H-2′′′,6′′′), 7.53 (d,* J* = 7.9 Hz, 2H, H-2′,6′), 7.44 (m, 1H, H-4′′′), 7.20 (m, 2H, H-3′′′,5′′′), 7.10 (d,* J* = 8.1 Hz, 1H, H-6), 7.02 (d,* J* = 7.8 Hz, 2H, H-3′,5′), 6.89 (s, 1H, H-8), 6.78 (d,* J* = 16.8 Hz, 1H, H-2′′), 3.85 (s, 3H, -OCH_3_). ^13^C-NMR (DMSO-*d*_6_) *δ* (ppm): 180.89 (C-4), 169.59 (C-1′′), 163.31 (C-7), 158.32 (C-4′), 157.18 (C-8a), 154.42 (C-2), 148.91 (C-3′′), 136.49 (C-1′′′), 131.39 (s, C-4′′′), 130.44 (s, C-2′,6′), 129.48 (C-3′′′,5′′′), 129.04 (C-2′′′,6′′′), 128.12 (C-5), 126.22 (C-1′), 124.18 (C-3), 118.38 (C-2′′), 116.89 (C-6), 115.48 (C-8), 114.32 (C-3′,5′), 105.32 (C-4a), 57.52 (-OCH_3_). HRMS (ESI)* m*/*z*: 399.10322 [M + H]^+^, calcd. for C_25_H_19_O_5_ 399.12325.

*(6) Synthesis of Compound* ***6***. A methanolic solution (30.00 mL) of VO(acac)_2_ (0.27 g, 1.018 mmol) was added to a methanolic solution (20.00 mL) of genistein (0.54 g, 1.998 mmol). The mixture was stirred at room temperature for 30 min to produce a deep-brown solution. This solution was allowed to stand in air for a few days until three-quarters of the solvent had evaporated. The crystals were isolated via filtration, washed three times with cold methanol, and air-dried.

*Physicochemical Properties and Elemental Analysis*. The genistein-vanadium complex was in the form of a brown powder that could be dissolved in methanol, acetone, or THF. Its element contents are shown in Table 1. The content of vanadium was determined via inductively coupled plasma (ICP) atomic emission spectrometry.

*Thermogravimetry (TG) and Differential Thermal Analysis (DTA)*. Based on TG and DTA, the main weight loss process occurred at 330°C~410°C (the heating rate was 10°C/min), and the weight loss rate was 88.91%. This weight loss rate was similar to the theoretical weight loss rate for two ligands (88.94%).

**Figure 3:**
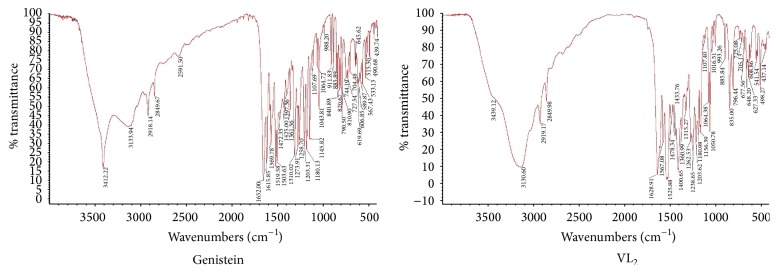
IR spectrum analysis.

*Infrared Spectrum Analysis*. The main infrared data are shown in Figure 3 and Table 2.

The absorption peak of the hydroxyl was shifted from 3412 cm^−1^ (genistein) to 3439 cm^−1^ (complex). The absorption peak of the carbonyl was shifted from 1652 cm^−1^ (genistein) to 1629 cm^−1^ (complex). Therefore, a 4-carbonyl group is involved in the coordination of the ligands.

**Figure 4:**
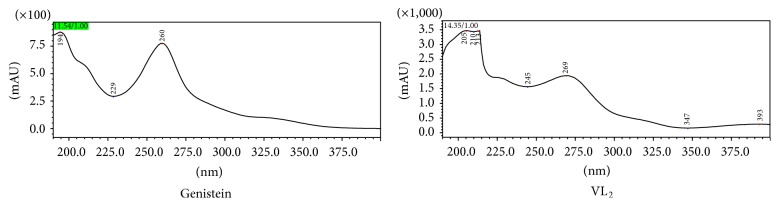
UV spectrum analysis.

*Ultraviolet Spectrum*. In the DMSO : CH_3_OH solvent (1 : 10), two characteristic UV absorption peaks were red-shifted from 260 nm (band II, genistein) and 332 nm (band I, weak, genistein) to 269 nm (band II, complex) and 393 nm (band I, weak, complex), respectively. Upon complex formation, the 5-hydroxyl, 4-carbonyl, and vanadium elements formed a coordination structure. The molecular flatness was increased, as the B ring more effectively formed a conjugate structure with the C ring. Thus, bands I and II were red-shifted, and the intensity of these bands was increased. These results are shown in Figure 4.

#### 2.2.2. Biological Activity

*(1) Hypoglycemic Activity Screening*. Cell culture and IR-HepG2 cell model [31–33]: HepG2 cells were recovered with high-glucose Dulbecco's modified Eagle's medium (DMEM) containing 10% inactivated fetal bovine serum (FBS) and 1% penicillin-streptomycin. The cells were cultured at 37°C and 5% CO_2_ in an incubator. When the cells were attached to the wall and became more than 80% confluence, the culture medium was discarded. The adherent cells were digested using 0.25% trypsin and then was diluted 1 : 3 to go on culturing. Cells in the logarithmic growth phase were used for the experiment.

The cells were digested using 0.25% trypsin, and then the cell suspension was then diluted to a cell density of 5 × 10^4^ /mL using high-glucose medium containing 10% FBS and 1% penicillin-streptomycin. Next, 200 *μ*L of the diluted cell suspension was incubated in each well of a 96-well plate. When the cells were attached to the wall and became more than 80% confluence, the medium in each well was replaced with freshly prepared high-glucose DMEM containing 5 × 10^−7^ mol/L insulin to establish the cell model of insulin-resistance. After 24 h of stimulation with a high concentration of insulin, the cells were washed twice with PBS. Then, serum-free high-glucose DMEM supplemented with compounds at different concentrations was added. After 24 h of incubation at 37°C and 5% CO_2_ in an incubator, the glucose concentration in the culture medium was assessed using a glucose assay kit. Glucose consumption (GC) in the cell culture medium was calculated for each group. The formulas for this calculation are as follows: glucose content (mmol/L) = (absorbance of each well/absorbance of calibration solution) × concentration of calibration solution, GC = glucose concentration in the cell culture medium without cells- glucose concentration in the cell culture medium of each group. Finally, cell viability was measured using a cck-8 kit. Add 10 *μ*L of CCK-8 solution to each well (be careful not to create bubbles in the wells, which will affect OD readings) and then incubate the plates in the incubator for 1–4 hours. Measure the absorbance at 450 nm with a microplate reader. CCK8 is the absorbance at 450 nm with a microplate reader. So GC/CCK8 is the ratio of the amount of glucose consumed by cells to the amount of viable cells. The meaning of GC/CCK8 is to avoid differences in sugar consumption by avoiding differences in cell densities, thus avoiding false positive or false negative results.

Compounds** 1b**,** 3c**,** 4a**, and** 6** and genistein were selected for combination. These compounds displayed higher hypoglycemic activity than the other derivatives. Three compounds were mixed to form each combination. The concentration of these compounds used for testing was 1/3 of the original concentration. This concentration was determined primarily based on our previous study [31], and the content of genistein, biochanin A, and formononetin in chickpeas was approximately 1 : 1 : 1.

*(2) Effects of Combination 3 and Combination 6 on H4IIE Cells*

*Cell Culture*. H4IIE cells were recovered with the complete medium. The cells were cultured in an incubator at 37°C and 5% CO_2_. When the cells are attached to the wall and become more than 80% confluent, the medium is discarded. Adherent cells were digested with 0.25% trypsin and then diluted at 1 : 3, and the culture was continued. Cells in logarithmic growth phase were used for experiments.

*CCK-8 Assay*. H4IIE cells at logarithmic growth phase were seeded into 96-well plates and incubated at 37°C in an atmosphere of 5% CO_2_ in the incubator. After the cells reach confluence, the cells were cultured in serum-free medium (blank control group), serum-free medium with metformin (metformin group), and serum-free medium with sample (sample intervention group). After going on incubating for 24 hours in 37°C incubator, 10 *μ*L of CCK8 was added into each well and then incubated in 37°C incubator for 1 h. Place the plates in the microplate reader detection, the wavelength is set to 450 nm, and measure absorbance values. The survival rate % = $\overset{-}{A}/{\overset{-}{A}}_{0}\times 100\%$, wherein $\overset{-}{A}$ was the average absorbance value of metformin group or sample intervention group, and ${\overset{-}{A}}_{0}$ was the average absorbance value of blank control group.

Research has shown that the concentration of resistin in obese rats was approximately 60 ng/mL [34]. An excessive resistin concentration may change the activity of liver cells, and this alteration interferes with the objective assessment of the function of hepatocytes. Previous results [34] also showed that stimulating H4IIE hepatocytes with 100 ng/mL (>60 ng/mL) resistin for 8 h produced no significant changes in the activity of H4IIE cells compared to the blank control treatment without resistin. This result indicated that the activity of H4IIE hepatocytes would not be significantly affected by resistin at 100 ng/mL. Therefore, in this study, 60 ng/mL of resistin was used as the stimulatory concentration in liver cells.

Measurement of cellular glycogen cotent:

1. When the cells had grown to 80% confluence, they were washed twice with physiological saline and then cultured in serum-free medium for 12 h.
2. The glucose concentration of the medium was adjusted to 4.5 g/L.
3. Experimental grouping is as follows: blank control group (group 1): did not add resistin or test compounds; negative control group (group 2): added resistin but did not add test compounds; test group (group 3): added resistin and combinations of test compounds (combination 3 and combination 6); and positive control group (group 4): added resistin and rosiglitazone hydrochloride (positive control drug). The above groups were divided into two conditions: with and without insulin stimulation.
4. Concentration and time of stimulation are as follows: after 2 h of pretreatment with resistin (60 ng/mL), insulin (100 nM) was added for 2 h.
5. The supernatant was discarded, and the cells were washed twice with cold physiological saline. Next, 100 *μ*L of 30% KOH was added, and the cells were then scraped for retrieval. The cells were repeatedly frozen and thawed at −20°C and 4°C, respectively, to generate lysates.
6. The supernatant was boiled for 20 min and centrifuged at 4000 rpm for 15 min. The supernatant was precooled on ice.
7. Glucose standards (0.005, 0.01, 0.03, 0.05, 0.07, and 0.09 mg/mL) were prepared.
8. An aliquot of 2 mL of precooled anthrone reagent and 0.9 mL of precooled distilled water were added to the test samples and the standards (anthrone reagent: 2 g of anthrone was dissolved in 98% H_2_SO_4_ and diluted to 1000 mL with 98% H_2_SO_4_, and the solution was used on the day of preparation).
9. The solution was then fully mixed and boiled for 5 min in water. The absorbance of the liquid was measured at a wavelength of 620 nm after cooling.
10. Method of calculation is as follows: for this measurement, 1.11 was the coefficient at which the content of glucose was converted into glycogen content. In other words, the coloration of 100 *μ*g of glycogen complexed with anthrone reagent was equivalent to that of 111 *μ*g of glucose complexed with anthrone reagent.

#### 2.2.3. Statistical Analysis

The results were presented as mean ± SD, of which the mean was the average of at least three replicates and SD was the standard deviation. The data were analyzed by* t*-test using SPSS 22.0.

## 3. Results and Discussion

### 3.1. Synthesis

In this study, a series of new compounds were generated via structural modification. Among these derivatives, compounds** 1a**,** 1c–1d**,** 2a–2d**,** 3a–3d**,** 3f–3h**,** 4c**,** 4h**,** 5a–5c**, and** 6** are the new compounds that have not previously been reported in the literature. The structures of these compounds are shown in Table 3.

### 3.2. Biological Activity

**Figure 5:**
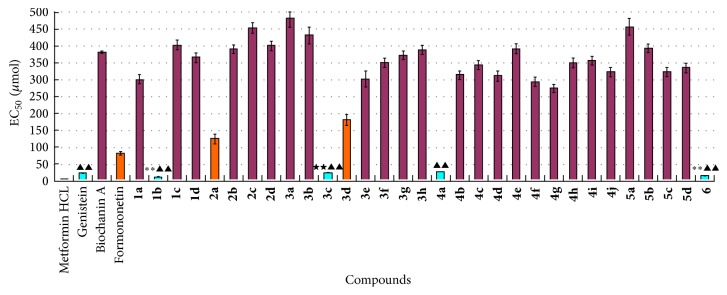
Hypoglycemic activity of the compounds ($\overset{-}{x}\pm s$, *n* = 3).

The hypoglycemic activity of these isoflavone derivatives and their parent compounds was screened using an IR HepG2 cell model [35–37]. Among them, compounds** 1b**,** 3c**,** 4a,** and** 6** displayed clearly good hypoglycemic activity. Genistein also showed good hypoglycemic activity in these cells. The hypoglycemic activity of each compound is shown in Table 4 and Figure 5.

In this study, the activity of the compounds was stratified into three levels: EC_50_ < 30.0 *μ*mol; 200.0 *μ*mol > EC_50_ > 30.0 *μ*mol; and EC_50_ > 200.0 *μ*mol. These three levels were considered to represent strong, moderate, and no hypoglycemic activity and are shown in Figure 5 in bright blue, orange, and purple, respectively. Compounds** 1b** and** 6** displayed higher hypoglycemic activity than genistein (parent compound) (“*∗∗*” denotes *p* < 0.01). Compound** 3c** displayed higher hypoglycemic activity than biochanin A (parent compound) (“★★” denotes *p* < 0.01). Compound** 4a** displayed considerable hypoglycemic activity that was comparable to that of genistein (*p* > 0.05). However, in general, these derivatives displaying high hypoglycemic activity, including the parent compound genistein, were still significantly less effective than the positive control drug metformin hydrochloride (“▲▲” denotes *p* < 0.01).

*Structure-Activity Relationship Analysis*. Following condensation of DCC with acetyl isoferulic acid, hypoglycemic activity was increased only when 4′,7-acetyl isoferulic acid was introduced simultaneously. Compared with genistein, the condensed compounds showed significantly different hypoglycemic activity (*p* < 0.01), and their hypoglycemic activity was reduced if only 7-acetyl isoferulic acid was introduced to the compounds. This result demonstrated that the strength of binding to the target could be enhanced by the addition of more acetyl isoferulic acid.

Following condensation with L-aspartic acid dimethyl ester, regardless of whether 7-L-aspartic acid dimethyl ester was introduced alone or simultaneously with 4′,7-di-L-aspartic acid dimethyl ester, the hypoglycemic activity of the compounds was significantly decreased.

Following phosphorylation of the isoflavone, compound** 3c** displayed significantly higher hypoglycemic activity than biochanin A (*p* < 0.01). The hypoglycemic activity of the other compounds was decreased or not significantly altered by phosphorylation. The hypoglycemic activities of compounds** 3c** and** 3g** were compared, showing that the compound did not require a large lipid group. Moreover, the hypoglycemic activities of compounds** 3c** and** 3a** were compared, showing that the hypoglycemic activity when 4′-H was substituted by a methoxyl group was superior to that when 4′-H was substituted by a hydroxyl group under 7-H which was substituted by diethyl phosphite.

For the bromine alkylation of genistein, biochanin A, and formononetin, compound** 4a** displayed considerable hypoglycemic activity that was comparable to that of genistein (*p* > 0.05). Compound** 4d** displayed significantly higher hypoglycemic activity than biochanin A (*p* < 0.05). Compounds** 4d** and** 4e** were compared, showing that the hypoglycemic activity when 5-H was substituted by hydroxyl was superior to that when 5-H was not substituted under 7-H which was substituted by a 2-bromo-ethoxy group (*p* < 0.05). The hypoglycemic activities of compounds** 4e** and** 4j** were compared, showing that 7-H should be substituted by a shorter alkyl bromide (*p* < 0.05).

For DCC condensation with cinnamon acid, compound** 5c** displayed significantly higher hypoglycemic activity than biochanin A (*p* < 0.05).

Following the formation of vanadium-containing complexes, the hypoglycemic activity of compound 6 was increased (*p* < 0.05). This result demonstrated that formation of vanadium-containing complexes increased the hypoglycemic activity of the compounds.

The results of structure-activity relationship analysis are shown in Table 5.

**Figure 6:**
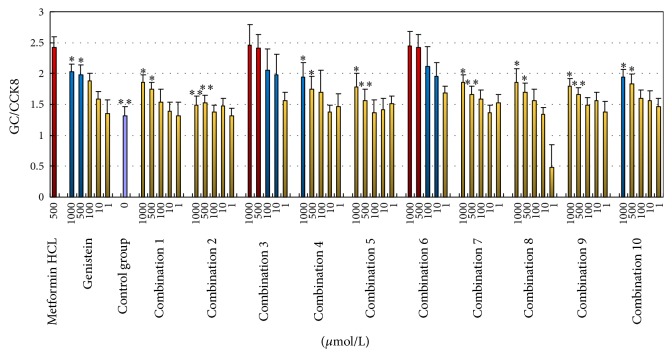
The hypoglycemic activity of the generated combinations ($\overset{-}{x}\pm s$, *n* = 3).

Based on the screen of the hypoglycemic activity, derivatives** 1b**,** 3c**,** 4a,** and** 6** showed better hypoglycemic activity. These 4 compounds and genistein (G, also showing good hypoglycemic activity) were mixed to form combinations. The hypoglycemic activity of the generated combinations is shown in Figure 6.

As observed from Figure 6, the hypoglycemic activity of combination 3 and combination 6 was superior to that of the other combinations and of genistein (“*∗*” and “*∗∗*” denote *p* < 0.05 and *p* < 0.01, resp.). The hypoglycemic activity of these two combinations was similar to that of the positive control drug (metformin hydrochloride), and at the same dose (500 *μ*mol/L), the difference was not statistically significant (*p* > 0.05).

Figures 6 and 5 showed that the actual hypoglycemic activity of these combinations was distinct from the accumulation of the activity of each individual compound. Figure 6 showed that combination 3 and combination 6 had the highest hypoglycemic activity. However, it can be calculated from the data of Figure 5 that combination 5 and the combination 9 had the highest hypoglycemic activity. This result showed that the combination of compounds would have a mutual impact. By acting on different targets simultaneously, the combinations produced higher hypoglycemic activity than each individual compound.

**Figure 7:**
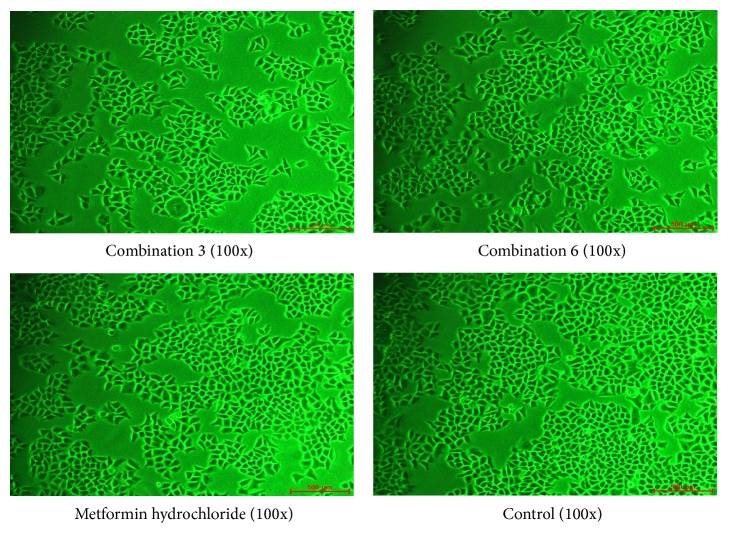
Cellular morphology.

Compared with the control group, the combined compounds produced no changes in cell morphology or cell number. These observations indicated that the toxicity of these combinations was low, as shown in Figure 7. And the survival rates of H4IIE are shown in Table 6.

**Figure 8:**
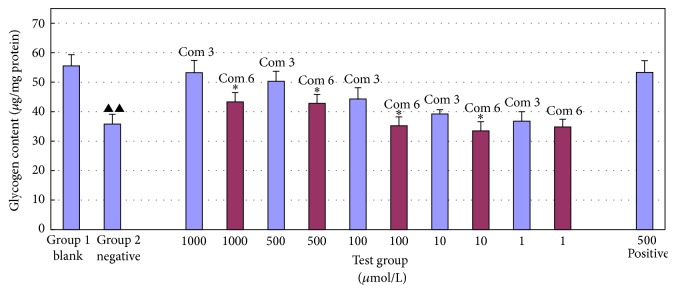
Glycogen metabolism without insulin stimulation of combination 3 and combination 6 ($\overset{-}{x}\pm s$, *n* = 3).

**Figure 9:**
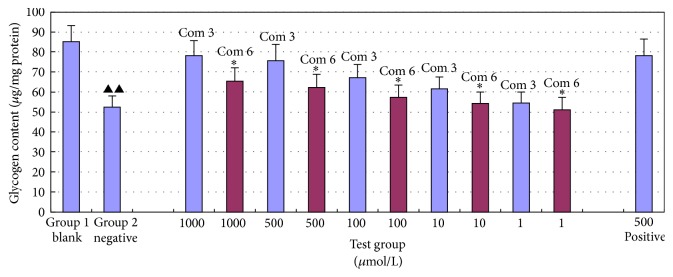
Glycogen metabolism with insulin stimulation of combination 3 and combination 6 ($\overset{-}{x}\pm s$, *n* = 3).

Serum-starved H4IIE hepatocytes were treated with resistin (60 ng/mL) for 2 h prior to insulin stimulation (100 nM for 2 h) or no stimulation. Afterwards, the glycogen content was measured. These results are shown in Figures 8 and 9.

As shown in Figure 8, comparing combination 3 and combination 6 in the absence of insulin stimulation showed that combination 3 was significantly superior in promoting glycogen synthesis (*p* < 0.05, denoted by “*∗*”). The effects of combination 3 at doses of 1000 and 500 *μ*mol/L (the same dose as the positive control drug) were identical to those of the positive drug; the difference in glycogen metabolism was not significant between these treatments (*p* > 0.05). In addition, the amount of glycogen was similar to that produced under normal conditions (*p* > 0.05). The difference was significant between groups 1 and 2 (*p* < 0.01, denoted by “▲▲”). As shown in Figure 9, under insulin stimulation, the effect of combination 3 was significantly superior to that of combination 6 (*p* < 0.05, denoted by “*∗*”). The effects of combination 3 at doses of 1000 and 500 *μ*mol/L (the same dose as the positive control drug) were identical to those of the positive control drug; the difference in glycogen metabolism was not significant between these treatments (*p* > 0.05). In addition, for combination 3, the amount of glycogen was similar to that produced under normal conditions (*p* > 0.05), indicating that combination 3 would have better hypoglycemic activity than combination 6 in future studies. The difference between groups 1 and 2 was significant (*p* < 0.01, denoted by “▲▲”).

## 4. Conclusion

In this study, our synthesized substrates were derived from isolates of chickpea, an Uygur food that possesses potential hypoglycemic activity. A series of derivatives were synthesized and their hypoglycemic activities were screened. Compounds** 1b**,** 3c**,** 4a**,** 6,** and genistein were combined to produce a greater hypoglycemic effect. At the cellular level, the combination 3 and combination 6 had similar hypoglycemic effects to the positive control drug (*p* > 0.05). These results indicate that these combinations of multiple compounds also act on many different targets, which can produce better results and greater hypoglycemic effects than any single compound.

In recent years, resistin was identified as a candidate insulin resistance molecule secreted by fat cells. Current evidence suggests that resistin represents a link between obesity and type 2 diabetes. H4IIE cells were cultured in vitro and treated with recombinant resistin protein. The amount of glycogen was measured under conditions of insulin stimulation and baseline conditions (no insulin stimulation) to evaluate the changes in the insulin sensitivity of liver cells. Based on this study, combination 3 was superior to combination 6 (*p* < 0.05) in increasing the sensitivity of liver cells to insulin. This result may provide a basis for the discovery of new hypoglycemic drugs and new drug delivery systems.

Further biological evaluations and investigations into the mechanisms of action of the generated active compounds are currently underway.

## Figures and Tables

**Table 1:** Elemental analysis of the genistein-vanadium complex.

C%		H%		V%		Molecular weight	
Test	Theory	Test	Theory	Test	Theory	Test	Theory [M − H]^−^
59.50	59.52	3.02	3.00	8.44	8.41	604.02131	604.02105

**Table 2:** Primary IR spectral data of genistein and the genistein-vanadium complex (cm^−1^).

Compound	*ν* (O-H)	*ν* (C=O)	*ν* (C=C)	*ν* (C-O-C)
Genistein (L)	3412	1652	1616	1259
VL_2_	3439	1629	1611	1263

**Table 3:** Structures of the generated compounds.

Compounds	R	R′	R′′
**1a**	-OH	-H	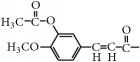

**1b**	-OH	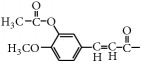	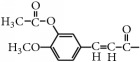

**1c**	-OH	-CH_3_	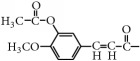

**1d**	-H	-CH_3_	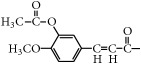

**2a**	-OH	-H	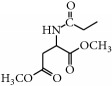

**2b**	-OH	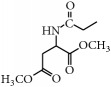	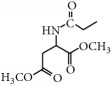

**2c**	-OH	-CH_3_	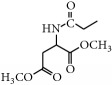

**2d**	-H	-CH_3_	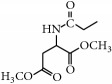

**3a**	-OH	-H	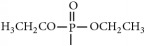

**3b**	-OH	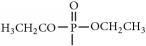	-H

**3c**	-OH	-CH_3_	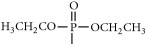

**3d**	-H	-CH_3_	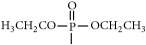

**3e**	-OH	-H	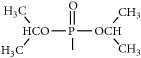

**3f**	-OH	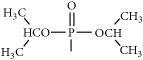	-H

**3g**	-OH	-CH_3_	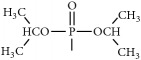

**3h**	-H	-CH_3_	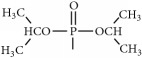

**4a**	-OH	-H	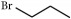

**4b**	-OH	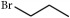	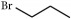

**4c**	-OH	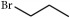	-H

**4d**	-OH	-CH_3_	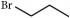

**4e**	-H	-CH_3_	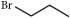

**4f**	-OH	-H	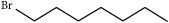

**4g**	-OH	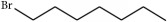	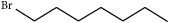

**4h**	-OH	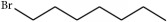	-H

**4i**	-OH	-CH_3_	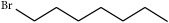

**4j**	-H	-CH_3_	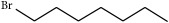

**5a**	-OH	H	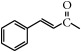

**5b**	-OH	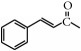	H

**5c**	-OH	-CH_3_	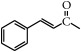

**5d**	-H	-CH_3_	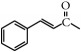

R = OH or H, and this group did not participate in the reaction.

**Table 4:** Effects on glucose consumption of these derivatives in IR HepG2 cells (*n* = 3).

Compounds	*n*	EC_50_	Standard deviation
Metformin HCL	3	3.332	1.212
Genistein	3	22.212	1.112
Biochanin A	3	380.234	3.652
Formononetin	3	80.922	5.218
**1a**	3	300.221	13.221
**1b**	3	10.238	1.121
**1c**	3	401.588	14.322
**1d**	3	365.789	14.421
**2a**	3	123.981	13.921
**2b**	3	390.092	13.221
**2c**	3	452.289	15.682
**2d**	3	399.563	14.532
**3a**	3	482.229	25.685
**3b**	3	431.135	25.421
**3c**	3	23.678	1.331
**3d**	3	178.981	15.789
**3e**	3	300.982	24.001
**3f**	3	349.231	13.562
**3g**	3	372.221	13.862
**3h**	3	387.811	13.951
**4a**	3	392.381	14.562
**4b**	3	312.811	13.023
**4c**	3	342.873	13.465
**4d**	3	310.382	13.891
**4e**	3	24.981	1.223
**4f**	3	292.893	13.562
**4g**	3	273.445	12.119
**4h**	3	348.934	14.558
**4i**	3	356.378	13.214
**4h**	3	321.663	13.456
**5a**	3	456.897	24.721
**5b**	3	392.734	14.113
**5c**	3	321.665	14.112
**5d**	3	333.991	13.821
**6**	3	13.934	1.223

**Table 5:** Structure-activity relationship analysis results.

Hypoglycemic activity	Structure-activity relationship	
	Active groups	Inert groups
Genistein > formononetin > biochanin A	4′-OH is a strong active group	4′-OCH_3_ and 5-OH were groups that prevented the production of strong activity
**1b** > Genistein > formononetin > **1a** > **1d** > biochanin A > **1c**	4′,7-Diacetyl isoferulic acid in genistein	7-Acetyl isoferulic acid in genistein, biochanin A, and formononetin
Genistein > formononetin > **2a** > biochanin A > **2b** > **2d** > **2c**	none	4′,7-di-L-Aspartic acid dimethyl ester in genistein; 7-L-aspartic acid dimethyl ester in genistein, biochanin A, and formononetin
Genistein > **3c** > formononetin > **3d** > **3e** > **3f** > **3g** > biochanin A > **3h** > **3b** > **3a**	7-Diethyl phosphite in biochanin A	7-Diethyl phosphite in genistein, and formononetin; 4′-diethyl phosphite in genistein; 7-diisopropyl phosphite in genistein, biochanin A, and formononetin; 4′-diisopropyl phosphite in genistein
Genistein > **4a** > formononetin > **4g** > **4f** > **4d** > **4b** > **4j** > **4c** > **4h** > **4i** > biochanin A > **4e**	7-2-Bromo-ethoxy in biochanin A	7,4′-Di-2-bromo-ethoxy or 7-2-bromo-ethoxy or 4′-2-bromo-ethoxy in genistein; 7-2-bromo-ethoxy in formononetin;
		7,4′-Di-6-bromo-hexyloxy or 7-6-bromo-hexyloxy or 4′-6-bromo-hexyloxy in genistein; 7-6-bromo-hexyloxy in biochanin A; 7-6-bromo-hexyloxy in formononetin
Genistein > formononetin > **5c** > **5d** > biochanin A > **5b** > **5a**	7-Cinnamic acid in biochanin A	7-Cinnamic acid in genistein, and formononetin; 4′-cinnamic acid in genistein
**6 **> Genistein > formononetin > biochanin A	V^2+^	

**Table 6:** Survival rates of H4IIE cells treated with the combinations and metformin ($\overset{-}{x}\pm s$, *n* = 6).

	Metformin HCL	Combination 3	Combination 6
Survival rate (%)	95.56 ± 2.16	94.47 ± 0.92	95.41 ± 0.55
